# Saturation kinetics of iodine accumulation in *Tenebrio molitor* (Coleoptera: Tenebrionidae): implications for feed biofortification

**DOI:** 10.1093/jisesa/ieag098

**Published:** 2026-09-19

**Authors:** Stepan Ryba, Yupa Hanboonsong, Jana Prodelalova, Martin Kulma, Hung Quang Tran, Hai Thanh Duong, Nguyen Phuc Cam Tu, Vlastimil Stejskal

**Affiliations:** Faculty of Agriculture and Technology, University of South Bohemia, Ceske Budejovice, Czech Republic; Faculty of Agriculture, Department of Entomology, Khon Kaen University, Khon Kaen, Thailand; Department of Infectious Diseases and Preventive Medicine, Veterinary Research Institute, Brno, Czech Republic; Department of Zoology and Fisheries, Czech University of Life Sciences Prague, Praha-Suchdol, Czech Republic; Faculty of Fisheries and Protection of Waters, South Bohemian Research Center of Aquaculture and Biodiversity of Hydrocenoses, Institute of Aquaculture and Protection of Waters, University of South Bohemia in Ceske Budejovice, Ceske Budejovice, Czech Republic; Faculty of Animal Sciences and Veterinary Medicine, University of Agriculture and Forestry, Hue University, Hue, Vietnam; Department of Aquatic Biology and Resources Management, Nong Lam University, Ho Chi Minh City, Vietnam; Faculty of Fisheries and Protection of Waters, South Bohemian Research Center of Aquaculture and Biodiversity of Hydrocenoses, Institute of Aquaculture and Protection of Waters, University of South Bohemia in Ceske Budejovice, Ceske Budejovice, Czech Republic

**Keywords:** bioaccumulation factor, excretion, fortification, insect nutrition, iodine toxicity

## Abstract

Iodine deficiency remains a widespread nutritional concern, and biofortification of feed ingredients is a promising complement to salt iodization. Insects, such as the yellow mealworm, *Tenebrio molitor* Linnaeus (Coleoptera: Tenebrionidae), are gaining attention as sustainable protein sources, yet little is known about their capacity to accumulate iodine. This study quantified dose-dependent iodine accumulation and excretion, and assessed adverse effects, in *T. molitor* larvae reared on diets spanning more than 3 orders of magnitude of supplemented iodine; saturation kinetics here refers to dose-dependent rather than temporal behavior. Larval iodine increased with supplementation but followed a strongly non-linear trend, a pattern compatible with saturable, carrier-mediated uptake, although no direct mechanistic evidence was obtained. Within the range in which larvae remained viable, accumulation conformed to a saturation model, allowing the half-saturation constant and maximum attainable enrichment to be estimated; at higher doses, larvae did not survive and could not be depurated. Frass iodine rose far more steeply than larval iodine, indicating that surplus iodine was excreted rather than retained. Bioaccumulation efficiency declined progressively with dietary dose, exceeding unity only at low supplementation levels, where larvae concentrated iodine above dietary concentrations. Observational signs of toxicity emerged at elevated supplementation, including growth retardation, heterogeneous development, and increased mortality, culminating in complete population collapse. Practically, the enrichment achievable within the range tolerated by larvae already exceeds concentrations reported as toxic to some target species, indicating that safe inclusion levels will be set by the receiving animal rather than the insect’s accumulation capacity.

## Graphical abstract

**Graphical Abstract:**
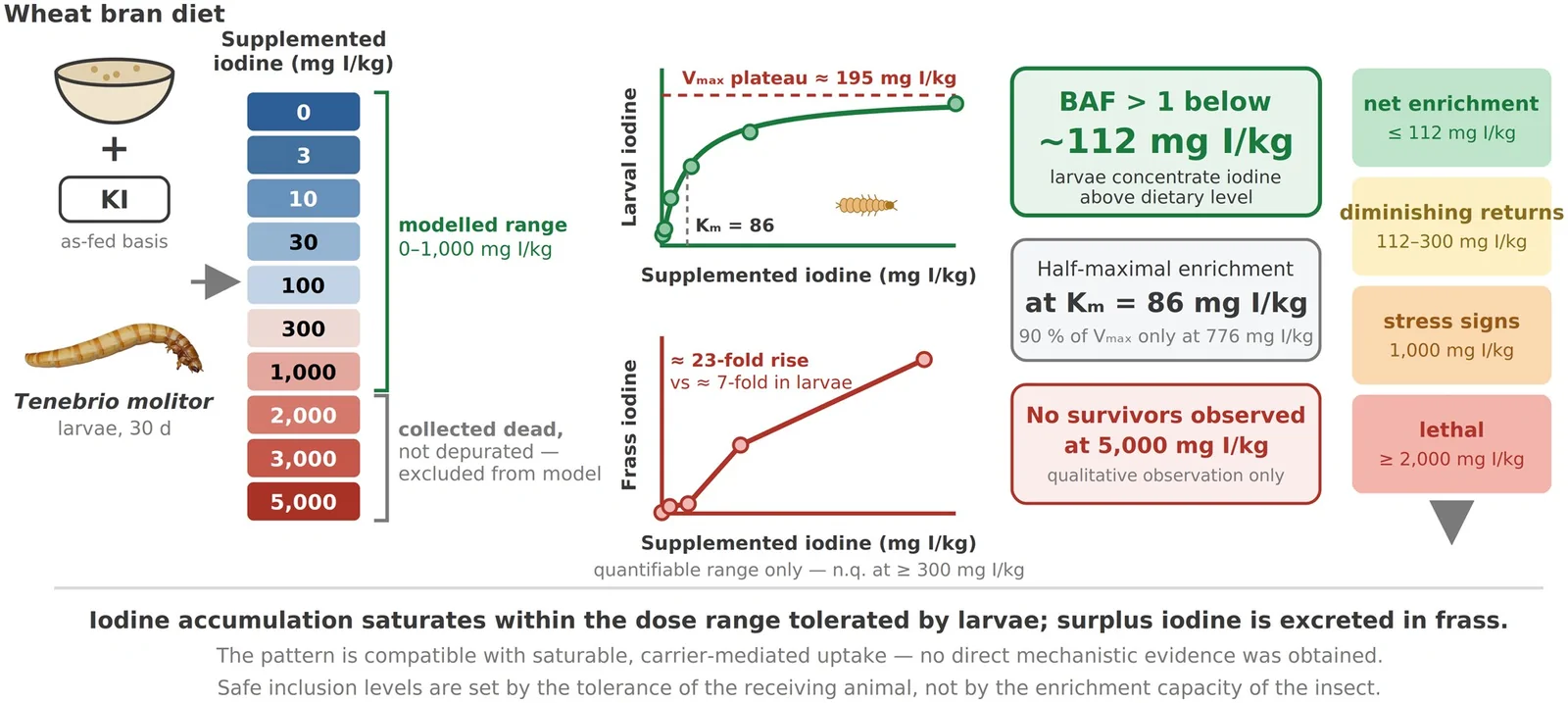

## Introduction

The primary strategy to combat iodine deficiency, a persistent global health issue, has been the iodization of salt. However, as public health initiatives advocate reduced salt intake, the importance of alternative iodine sources in the food chain is increasing (Van Der Reijden et al. 2017). Biofortification, the deliberate enhancement of the micronutrient content of food or feed during production—whether through agronomic practice, plant breeding, or dietary supplementation of the producing organism—represents a key complementary strategy. Unlike post-harvest fortification, in which the nutrient is added to the final product, biofortification incorporates the nutrient into the biological matrix itself, which can improve its stability and bioavailability. This approach has been successfully applied to enhance the content of iodine, selenium, and other essential elements in various plants (Gonzali et al. 2017).

Building on this principle, attention has turned to the biofortification of insects, which are increasingly recognized as a sustainable and nutritionally valuable protein source for animal feed, particularly in aquaculture and poultry farming. The yellow mealworm, *Tenebrio molitor* L. (Coleoptera: Tenebrionidae), offers significant environmental and economic advantages, including high feed conversion efficiency, minimal water and land requirements, and the ability to be reared on organic by-products (Makkar et al. 2014; Lienhard et al. 2023). The concept of enriching insect biomass is not new; studies have demonstrated the successful bioaccumulation of zinc and selenium in species like *T. molitor* and the black soldier fly, *Hermetia illucens* (L.) (Diptera: Stratiomyidae) (Ferrari et al. 2022; Nielsen et al. 2022; Machado et al. 2024; Copelotti et al. 2025). This parallels the well-established practice of fortifying conventional livestock feed with iodine to increase its concentration in animal products such as milk, meat, and eggs (Herzig et al. 2007; Flachowsky et al. 2014). Consequently, biofortified insect meal could serve as an effective “delivery vehicle” for iodine and other trace minerals within animal feed value chains. This is particularly relevant for aquaculture, where iodine plays a critical role in the health and development of fish larvae (Peng et al. 2024).

Despite the growing body of research on insect biofortification with trace elements, the metabolic and bioaccumulation dynamics of iodine in *T. molitor* remain largely uncharacterized. Three questions are unresolved: how efficiently larvae absorb and retain iodine across a range of dietary concentrations; whether absorption becomes saturated and at what level; and what happens to the fraction that is not absorbed.

Insects may handle iodine differently from both plants and vertebrates. They lack a thyroid gland, the organ that in vertebrates sequesters the majority of body iodine and thereby limits its distribution to other tissues (Franke et al. 2008). Whether the absence of this sink results in broader tissue distribution—and hence higher whole-body enrichment—has not been tested.

Without this knowledge, industrial-scale fortification risks being economically inefficient through wastage of non-absorbed iodine and potentially toxic to the insects themselves.

Therefore, this study was designed to address this gap. We hypothesized that iodine bioaccumulation in *T. molitor* larvae is not linear but saturable and that exceeding a threshold dietary concentration would reduce transfer efficiency and produce observable adverse effects.

This study differs from previous work on iodine in insects in 3 respects. First, earlier reports established that *T. molitor* can accumulate iodine but did so at a single or narrow range of dietary levels (EFSA Panel on Nutrition, Novel Foods and Food Allergens (NDA Panel) et al. 2023, Jankauskienė et al. 2024); here, dietary iodine was varied across more than 3 orders of magnitude (3 to 5,000 mg/kg), allowing the shape of the dose–response relationship to be characterized rather than assumed. Second, iodine was quantified in frass as well as in larvae, so that the fate of the non-absorbed fraction could be addressed directly. Third, the dose range extended into the toxic and lethal region, defining the upper boundary of what is biologically feasible rather than only what is nutritionally desirable.

The objectives were to: (i) quantify the relationship between dietary iodine and its concentration in larvae and frass; (ii) model the accumulation to characterize its non-linearity; (iii) determine how transfer efficiency varies with dose; and (iv) assess adverse effects of high iodine doses.

## Materials and Methods

### Animals and Rearing Conditions

All experimental procedures involving animals were conducted in accordance with applicable national legislation governing the care and use of animals for scientific purposes (Act No. 246/1992 Coll. on the Protection of Animals against Cruelty, Czech Republic) and in accordance with the permit issued to the University of South Bohemia in České Budějovice, Faculty of Agriculture and Technology (permit no. MZE-17931/2022-13143). The experimental protocol adhered to the welfare standards established for invertebrates used in nutritional research. Larvae of the yellow mealworm (*T. molitor* L.) were obtained from a commercial supplier (Papek s.r.o., Czech Republic). The larvae were maintained under controlled laboratory conditions, consistent with established rearing practices for this species (Kröncke et al. 2019). The rearing substrate consisted of wheat bran, and the colonies were kept in darkness in ventilated plastic containers at 27 ± 1 °C and 60% ± 5% relative humidity. To prevent desiccation and ensure adequate hydration, the larvae and rearing substrate were lightly misted with deionized water every second day.

### Experimental Design and Diets

The experiment was conducted to investigate the saturation kinetics of iodine bioaccumulation in *T. molitor* larvae. A basal diet was prepared from wheat bran and subsequently divided into 10 portions. Wheat bran was selected because it is the standard rearing substrate for *T. molitor* and is widely used in insect-nutrition studies, allowing comparability with published work; its intrinsic iodine content was below the limit of quantification of the analytical method (see below), so that dietary iodine could be attributed to the added supplement. One portion served as the control diet without added iodine. The other 9 portions were supplemented with analytical grade potassium iodide (KI; Penta s.r.o., Czech Republic) to achieve the following nominal iodine concentrations on an as-fed basis: 3; 10; 30; 100; 300; 1,000; 2,000; 3,000; and 5,000 mg I/kg. All dietary iodine concentrations are expressed on an as-fed basis throughout.

Larvae of *T. molitor* of uniform developmental stage were randomly distributed into rearing containers, with a total larval biomass of 200 g per container. Although individual instars were not determined, all larvae originated from the same cohort and were at a similar developmental stage, with none approaching pupation; the absence of instar determination represents a potential source of within-container variability. Each container received 400 g of the respective experimental diet at the start of the 30-day trial. Each dietary treatment was assigned to a single rearing container. The larvae were maintained on the diets for 30 days under ad libitum feeding conditions.

### Sample Collection and Preparation

At the end of the experimental period, larvae and their frass were separated from the feed substrate using a 1 mm sieve. At this stage, the remaining material consisted solely of frass, with no intact feed residues detected on visual inspection during sieving; this was not verified analytically. This observation indicated that the substrate had been completely consumed and re-ingested by the larvae during the final feeding phase. An exception was observed at the highest iodine concentrations (2,000 to 5,000 mg I/kg), where excessive iodine exposure led to substrate deterioration and the occurrence of dead larvae together with wet, aggregated mixtures of frass and unidentifiable feed residues. To minimize the presence of residual gut contents, larvae from each treatment container were held without feed for 24 h, a duration commonly used in mealworm and other insect nutritional studies. Following this starvation period, the larvae were euthanized by freezing at −20 °C for 24 h prior to lyophilization. Because alkaline mineralization converts all iodine species to iodide and the method determines total iodine, short-term frozen storage is not expected to affect the analytical result.

At the 3 highest supplementation levels (2,000 to 5,000 mg I/kg), extensive mortality precluded the 24-h depuration period; larvae from these treatments were collected dead from the substrate, and their iodine concentrations may therefore include residual gut contents and adhering frass.

From each container, 3 separate subsamples of larvae were collected and processed independently. These constitute within-container (technical) replicates and are reported as mean ± SD (*n* = 3).

Both the larval and frass samples were subsequently lyophilized (freeze-dried) to a constant weight to determine dry matter content. The dried samples were subsequently homogenized into a fine powder using a laboratory mill to ensure uniformity prior to chemical analysis. All powdered samples were stored in airtight containers at room temperature until analysis.

### Iodine Determination

Total iodine in feed, larval, and frass samples was determined spectrophotometrically using the Sandell–Kolthoff reaction with brucine as the terminating reagent (principle after Bednář et al. 1964), following the alkaline-mineralization protocol of Plzák et al. (2009). No validated protocol exists for insect matrices; the procedure established for soil and plant material was therefore adapted, and the calibration range was selected accordingly (0 to 150 µg I/l).

Prior to analysis, samples were subjected to alkaline mineralization to completely decompose the organic matrix and convert all iodine species to iodide. Sample masses ranged from 1.4 to 17.4 mg (typically 2 to 6 mg), and each homogenized sample was mixed with potassium carbonate, dried, and ashed in a muffle furnace at 250 °C for 1.5 h followed by 580 °C for 2 h. Sample masses were deliberately kept low so that mineralizate concentrations fell within the calibration range; where this was insufficient, mineralizates were further diluted 1:10 or 1:20 prior to analysis. The resulting ash was dissolved in deionized water and filtered.

The residue was suspended in 6 ml of deionized water, centrifuged, and a 2 ml aliquot of the clear supernatant was taken for analysis. Calibration standards were carried through the identical mineralization and dilution procedure, so that the extraction volume cancels between standards and samples.

Iodine determination was based on the catalytic effect of iodide on the reduction of cerium (IV) by arsenite. The decrease in absorbance of Ce^4+^ at 430 nm was monitored spectrophotometrically. The reaction was terminated by the addition of brucine, forming a colored complex with residual Ce^4+^.

Quantification was based on a single external calibration series prepared from KI standards over the range 0 to 150 µg I/l. For the reference method, the reported limit of detection (LOD) and limit of quantification (LOQ) are 2.6 and 11.7 µg I/l, respectively (Bílek et al. 2005). Matrix-specific LOD/LOQ and recovery were not determined for larval and frass matrices in this study (see “Limitations of the Present Study” section). Repeatability is reported as the coefficient of variation among the 3 subsamples of each treatment (0.8% to 15.1%).

Frass samples from treatments supplemented with ≥300 mg I/kg could not be quantified: the dilution required to bring the mineralizate within the calibration range (≥1:100) would have required an aliquot of 0.02 ml, beyond reliable pipetting accuracy. These values are reported as not quantifiable (n.q.) rather than as numerical estimates.

### Qualitative Assessment of Adverse Effects

Potential adverse effects of high-iodine diets were evaluated through qualitative observations conducted throughout the 30-day experimental period. Due to the nature of the mass-rearing setup, no quantitative mortality counts or individual growth measurements were performed.

Observations focused on the general condition of the larvae, including the occurrence of visibly dead or moribund individuals, apparent growth retardation, changes in activity or behavior (e.g. reduced movement or lethargy), and alterations of the feed substrate. At the highest iodine concentrations (3,000 and 5,000 mg I/kg), an increased occurrence of deteriorated substrate, characterized by moisture accumulation, aggregation of frass and feed residues, discoloration, and mold growth, was observed.

Under these conditions, dead larvae frequently adhered to moist substrate and frass, forming compact aggregates, which prevented reliable separation and quantitative mortality assessment. In addition, larvae were observed to die at various developmental stages, and cannibalism could not be ruled out. Therefore, toxicity-related outcomes in this study should be interpreted as qualitative and exploratory, providing indicative thresholds rather than precise dose–response relationships. Accordingly, all adverse-effect outcomes reported in the Results section should be interpreted as observational indicators of biological stress rather than precise quantitative toxicity metrics.

### Statistical Analysis

Data were summarized and visualized using the ggplot2 package (version 3.5.1) in R 4.5.1 (R Core Team). Treatment values are reported as mean ± SD of the 3 subsamples.

Because each dietary treatment was represented by a single rearing container, formal statistical comparison among treatments was not possible; the 3 subsamples per treatment quantify within-container and analytical variability only. Analyses therefore treat the dietary levels as the unit of observation. The monotonicity of the dose–response relationship was assessed by Spearman’s rank correlation across all 10 dietary levels.

Dose–response modeling was restricted to the range in which larvae remained viable and the 24-h depuration protocol could be applied (0 to 1,000 mg I/kg supplementation). At the 3 highest levels, larvae were collected dead and could not be depurated; these treatments were excluded from the model and are reported descriptively.

Within this range, a Michaelis–Menten saturation model was fitted by non-linear least squares to treatment means:


$${\left[I\right]}_{larvae}=C_{0}+V_{max}\;.\;D/(K_{m}+D)$$

where D is supplemented dietary iodine, and C_0_ is the measured iodine concentration in control larvae (3.71 mg/kg). C_0_ was fixed rather than estimated, so that the model describes diet-derived accumulation; larvae obtained from a commercial supplier carry a pre-existing body iodine burden that a model constrained through the origin cannot represent. The model was compared with a linear fit by Akaike’s Information Criterion (Akaike 1974). Confidence bands were obtained by Monte-Carlo simulation (4,000 draws) from the parameter variance–covariance matrix. The bioaccumulation factor (BAF) was derived analytically from the same model as BAF = [*C*_0_ + *V*_max_ · *D*/(*K*_m_ + *D*)]/*D*. Statistical significance of model terms was assessed at α = 0.05.

## Results

### Dose-Dependent Accumulation of Iodine in Larval Tissues

The biofortification of *T. molitor* larvae with iodine was effective, with the concentration of iodine in larval tissues increasing in relation to the level of iodine supplemented in the diet. The intrinsic iodine content of the unsupplemented wheat bran was below the limit of quantification, and dietary iodine is therefore expressed as supplemented iodine throughout. The baseline iodine concentration in larvae fed the control diet was 3.71 mg/kg. Across the 9 supplementation levels, from 3 to 5,000 mg/kg, larval iodine rose from 13.76 mg/kg to a maximum of 902.3 mg/kg (Table 1).

**Table:** Table 1. Iodine concentration in *T. molitor* larvae and frass, and calculated bioaccumulation factor (BAF), in relation to supplemented dietary iodine

Sample	Supplemented iodine (mg/kg)	Larval iodine (mg/kg)	CV (%)	Frass iodine (mg/kg)	BAF
**0 (control)**	0	3.71 ± 0.25	6.6	3.30 ± 0.11	n.a.^a^
**1**	3	13.76 ± 2.08	15.1	23.90 ± 1.07	4.59 ± 0.69
**2**	10	21.54 ± 2.20	10.2	33.80 ± 2.91	2.15 ± 0.22
**3**	30	61.43 ± 4.91	8.0	239.34 ± 12.64	2.05 ± 0.16
**4**	100	102.62 ± 4.02	3.9	538.70 ± 6.25	1.03 ± 0.04
**5**	300	147.29 ± 2.61	1.8	n.q.^b^	0.49 ± 0.01
**6**	1000	184.16 ± 3.10	1.7	n.q.	0.18 ± 0.00
**7**	2000	332.71 ± 5.04	1.5	n.q.	0.17 ± 0.00
**8**	3000	698.80 ± 37.43	5.4	n.q.	0.23 ± 0.01
**9**	5000	902.30 ± 7.46	0.8	n.q.	0.18 ± 0.00

a Not calculable; basal dietary iodine was below the limit of quantification (measured 0.1241 mg/kg).

b Not quantifiable: the dilution required to reach the calibration range (≥1:100) would have required an aliquot of 0.02 ml, beyond reliable pipetting accuracy.

Values are mean ± SD of 3 subsamples taken from a single rearing container per treatment. Larvae from samples 7 to 9 were collected dead and could not be depurated; these treatments were excluded from the dose–response model.

A Spearman’s rank correlation confirmed a strong, monotonic relationship between supplemented dietary iodine and larval iodine concentration (ρ = 1.00, *P* < 0.001, *n* = 10 dietary levels). This reflects consistency of rank order across the tested range and does not imply a linear relationship.

**Fig. 1.**
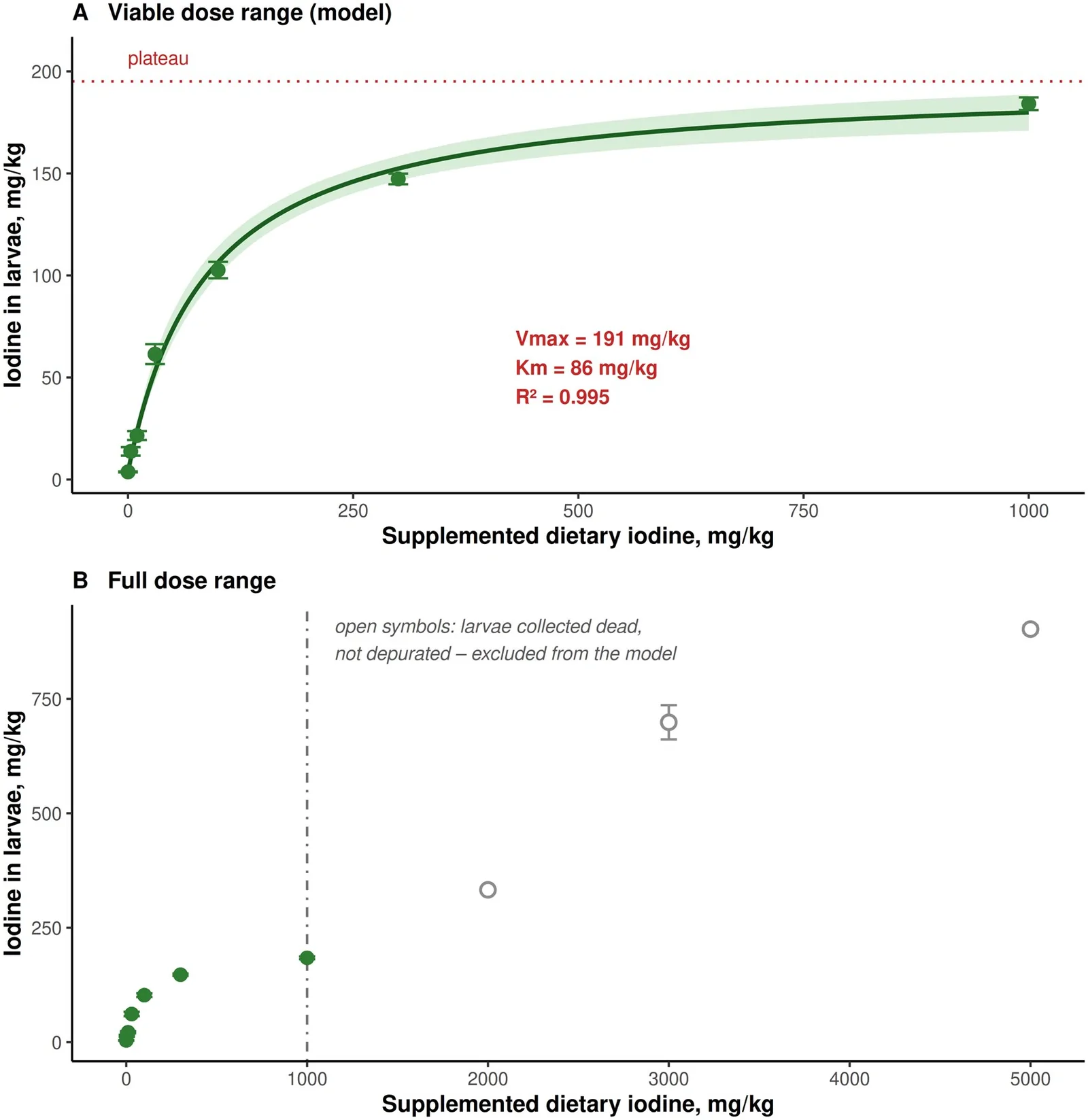
Iodine accumulation in *Tenebrio molitor* larvae in relation to supplemented dietary iodine. A) Viable dose range (0 to 1,000 mg I/kg), in which larvae survived and the 24-h depuration protocol was applied. Green circles show treatment means ± SD (*n* = 3 subsamples); the dark green curve is the fitted Michaelis–Menten model, [*I*]_larvae_ = *C*_0_ + *V*_max_ · *D*/(*K*_m_ + *D*), with C_0_ fixed at the measured control concentration (3.71 mg/kg). The shaded band is the 95% Monte-Carlo confidence interval, and the red dotted line marks the estimated plateau (*C*_0_ + *V*_max_ = 195.1 mg/kg). B) Full dose range. Open gray symbols denote the 3 highest supplementation levels, at which larvae were collected dead and could not be depurated; these treatments were excluded from the model. The dash-dotted line marks the upper limit of the modeled range.

Within the dose range in which larvae remained viable (0 to 1,000 mg I/kg), accumulation was strongly non-linear and closely followed Michaelis–Menten saturation kinetics (*R*^2^ = 0.995), substantially outperforming a linear fit (*R*^2^ = 0.712; ΔAIC = 28.5). The model estimated a maximum diet-derived enrichment of *V*_max_ = 191.4 ± 6.3 mg/kg, corresponding to a total plateau concentration of 195.1 mg/kg, with half-maximal accumulation at *K*_m_ = 86.2 ± 10.1 mg I/kg supplementation (Fig. 1A). Half of the attainable enrichment was therefore already reached at a supplementation level of approximately 86 mg/kg, and 90% at 776 mg/kg.

Larvae from the 3 highest supplementation levels reached nominally higher concentrations (332.7 to 902.3 mg/kg), but these animals were collected dead and could not be depurated; their values may include gut contents and adhering frass and were therefore excluded from the model (Fig. 1B, open symbols).

The saturating form of the relationship is consistent with a carrier-mediated absorption process operating at a finite capacity, although no direct evidence for such a mechanism is provided by the present data.

### Iodine Excretion in Frass Increases Steeply with Dietary Concentration

**Fig. 2.**
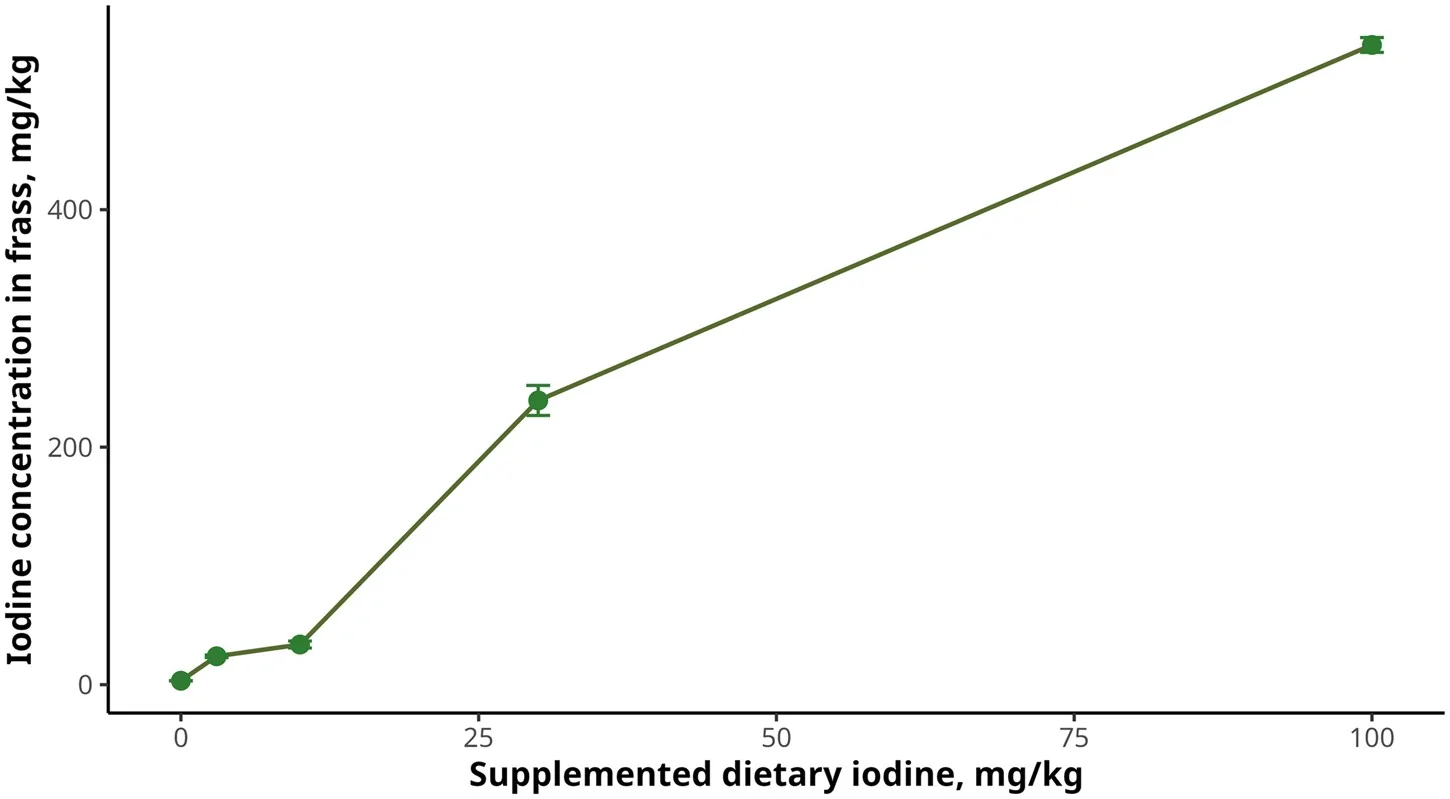
Iodine concentration in frass as a function of supplemented dietary iodine within the quantifiable range. Green circles show treatment means ± SD (n = 3 subsamples) for the 5 treatments in which frass iodine could be quantified; points are joined by a line to aid visual interpretation. No model was fitted, as only 5 dietary levels were available. Frass iodine at supplementation levels of 300 mg I/kg and above could not be quantified.

The concentration of iodine in the frass (excreta) produced by the larvae showed a markedly different trend compared to larval accumulation. While the control group exhibited a low frass iodine concentration of 3.30 mg/kg, this value increased sharply with dietary supplementation (Table 1). For supplementation levels within the quantifiable range (up to 100 mg/kg), frass iodine increased from 23.9 to 538.7 mg/kg (Fig. 2).

For supplementation levels of 300 mg/kg and above, frass iodine could not be quantified. Bringing these mineralizates within the calibration range would have required a dilution of at least 1:100, corresponding to an aliquot of 0.02 ml—beyond reliable pipetting accuracy at the volumes used. These values are therefore reported as n.q. rather than as numerical estimates and represent a methodological limitation rather than a physiological observation.

Within the quantifiable range, frass iodine rose approximately 23-fold, while larval iodine rose approximately 7-fold over the same interval, indicating that an increasing proportion of ingested iodine passed through the digestive tract unabsorbed (Baker 2004, Behmer 2009, Lind et al. 2023).

### Bioaccumulation Efficiency Declines with Increasing Dietary Iodine

To evaluate the efficiency of iodine transfer from the diet to the larvae, a BAF was calculated for each treatment as the ratio of larval iodine concentration to supplemented dietary iodine concentration:


$$\mathrm{BAF}\;=\;{\left[I\right]}_{\mathrm{larvae}}\;(\mathrm{mg}/\mathrm{kg})/{\left[I\right]}_{\mathrm{diet}}\;(\mathrm{mg}/\mathrm{kg})$$

BAF could not be calculated for the control treatment, as the basal dietary iodine concentration was below the limit of quantification.

**Fig. 3.**
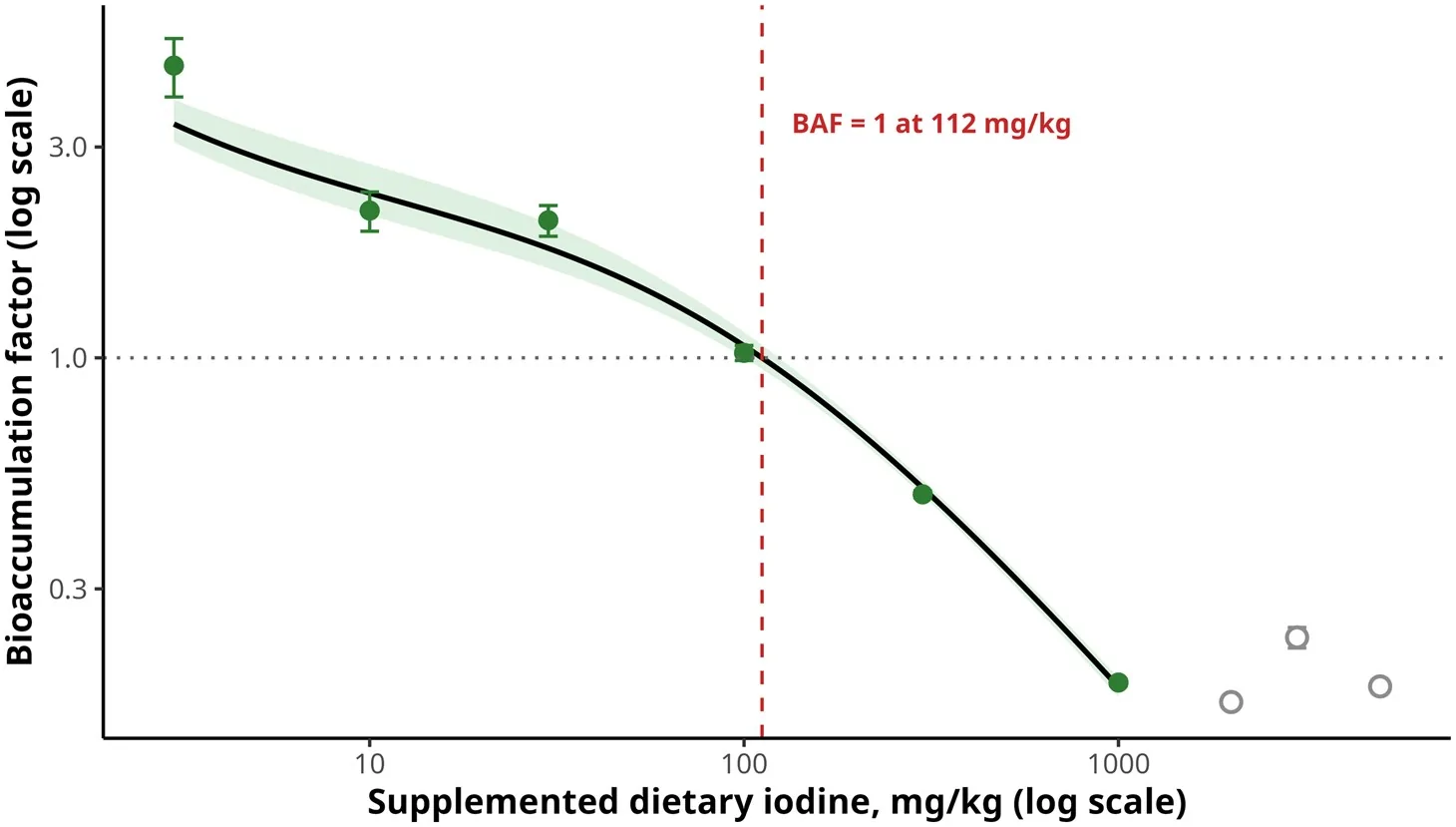
Bioaccumulation factor (BAF) in relation to supplemented dietary iodine, on logarithmic axes. Filled green circles show treatment means ± SD for the modeled dose range; open gray symbols denote the excluded treatments (larvae collected dead, not depurated). The black curve is derived analytically from the saturation model of Fig. 1A, BAF = (*C*_0_ + *V*_max_ · *D*/(*K*_m_ + *D*))/*D*, with the shaded band showing the 95% confidence interval. The horizontal dotted line marks BAF = 1, and the vertical dashed red line the dose at which the model crosses unity (112 mg I/kg), below which larvae concentrate iodine above dietary levels.

BAF declined monotonically across the tested range (Fig. 3). Derived from the fitted saturation model, BAF falls as [*C*_0_ + *V*_max_ · *D*/(*K*_m_ + *D*)]/*D* and passes unity at 112 mg I/kg. At the lowest supplementation levels, BAF exceeded unity—4.59 at 3 mg/kg and 2.05 at 30 mg/kg—indicating that larvae accumulated iodine to concentrations above those in their diet. Above the threshold, transfer efficiency fell progressively, to 0.49 at 300 mg/kg and 0.18 at 1,000 mg/kg. The model-derived threshold is consistent with observations, BAF being 1.03 at 100 mg/kg and 0.49 at 300 mg/kg.

The observed BAF at the lowest supplementation level (3 mg I/kg) exceeded the model prediction. This treatment had the highest coefficient of variation among all samples (15.1%) and the smallest sample masses, and at this dose, the pre-existing body iodine burden constitutes a large proportion of total larval content; the value should therefore be interpreted with caution.

This pattern identifies a practical threshold for biofortification: below approximately 112 mg I/kg supplementation, iodine is concentrated in larval biomass relative to the diet, whereas above it the majority of additional iodine is not incorporated into larval tissue and is instead excreted.

### High Dietary Iodine Concentrations Are Associated with Adverse Effects

Adverse effects were assessed by qualitative observation only. No quantitative mortality counts, survival rates, or individual growth measurements were recorded, and the outcomes reported below are observational indicators of biological stress rather than dose–response toxicity metrics.

Larvae in the control group and at supplementation up to 300 mg/kg showed no observable deviations in mortality or development.

Signs of stress first appeared at 1,000 mg/kg. In this group, the observed effects comprised visibly reduced growth relative to lower-dose containers, heterogeneous body size within the container indicating uneven development, and an increased number of dead individuals.

At, 2,000 and 3,000 mg/kg, a substantial number of dead larvae was observed, together with substrate deterioration—moisture accumulation, aggregation of frass and feed residues, discoloration and mold growth. Dead larvae frequently adhered to the moist substrate, preventing reliable separation and quantitative counting.

At 5,000 mg/kg, no surviving individuals were observed. Larvae from this treatment, and from the 2,000 and 3,000 mg/kg treatments, were collected dead and could not be depurated; their reported iodine concentrations may therefore include gut contents and adhering frass.

## Discussion

### An Integrated Physiological Response to Iodine Loading

This study provides the first quantitative characterization of the dose-dependent bioaccumulation, excretion, and toxicity of iodine in *T. molitor* larvae. Here, the term “kinetics” refers to dose-dependent saturation behavior rather than temporal uptake dynamics. The findings are consistent with a coherent physiological response to increasing dietary iodine. First, larval accumulation is dose-dependent but strongly non-linear, conforming within the viable dose range to a saturation model. Second, iodine in frass rises far more steeply than iodine in larvae. Third, transfer efficiency declines progressively with dose and falls below unity above a threshold that the model places at approximately 112 mg I/kg. Finally, adverse effects appear at high supplementation levels, culminating in population collapse.

These observations are best viewed not as independent phenomena but as interconnected stages of a single physiological continuum. Insects regulate the absorption of dietary minerals rather than absorbing them in proportion to intake (Behmer 2009), and dietary enrichment with 1 trace element has been shown to alter the handling of others in *T. molitor* (Keil et al. 2020). If intestinal uptake is saturable, surplus iodine would be expected to pass through the digestive tract, which is consistent with the steep rise in frass iodine. The decline in the BAF is the direct mathematical consequence of this dynamic: as larval accumulation approaches its plateau while the dietary concentration continues to increase, the ratio between them must fall. Adverse effects would then represent the point at which dietary load exceeds the capacity of this excretory route, although the present data do not establish this causally.

### Context of Iodine Bioaccumulation in Insects

The successful enrichment of *T. molitor* with iodine corroborates the broader principle that insects can serve as effective vectors for trace element biofortification, a concept previously established for selenium in *H. illucens* (Ferrari et al. 2022) and zinc in *T. molitor* (Keil et al. 2020). The present study extends prior research on iodine in insects. While an early study confirmed that *T. molitor* could accumulate iodine (EFSA Panel on Nutrition, Novel Foods and Food Allergens (NDA Panel) et al. 2023, Jankauskienė et al. 2024), it did not quantify the dose–response relationship or model the underlying kinetics. The present work provides a quantitative model of iodine accumulation across a wide dose range in this species, converting a qualitative observation into a description that can be tested and applied.

The saturable kinetics observed for iodine, an essential micronutrient, contrast with the mechanisms insects employ to manage non-essential toxic elements. For instance, *T. molitor* has been shown to actively deconcentrate cadmium by regularly shedding and excreting gut epithelial cells that have bound the metal, a strategy that prevents its systemic bioaccumulation (Van Der Fels-Klerx et al. 2016). This distinction suggests that *T. molitor* may possess a regulated, high-affinity transport system for absorbing a required nutrient, such as iodine, while using a different, bulk-excretion strategy to manage a non-essential toxin.

The transfer efficiencies observed here, which exceeded unity at low supplementation levels, are orders of magnitude higher than iodine carry-over rates reported for conventional livestock. For example, the transfer of supplemented iodine into pork muscle is exceptionally low, ranging from only 0.10% to 0.24%, and the overall transfer from feed into meat is generally below 1% (Franke et al. 2008). This difference in efficiency may be attributable to a fundamental physiological distinction: the absence of a thyroid gland in insects. In vertebrates, the thyroid acts as a highly efficient “iodine sink,” sequestering approximately 80% of the total body iodine to support hormone synthesis (Franke et al. 2008). This sequestration prevents the widespread distribution of iodine to other tissues, such as muscle. Lacking this specialized organ, insects may accumulate iodine more broadly throughout their body tissues, which would make the entire larval biomass a more concentrated vehicle for iodine biofortification.

### A Saturable, Carrier-Mediated Transport System

Within the range in which larvae remained viable, iodine accumulation conformed closely to Michaelis–Menten kinetics (*R*^2^ = 0.995), the functional form expected of a process limited by a finite number of membrane carriers. The estimated half-saturation constant, *K*_m_ = 86 mg I/kg supplemented iodine, provides a quantitative descriptor of this saturation, and the fitted *V*_max_ indicates a maximum attainable enrichment of approximately 195 mg/kg under the present conditions.

This remains a phenomenological description. No molecular, biochemical, or kinetic evidence for a specific transporter was obtained, and the fit is consistent with—but does not demonstrate—carrier-mediated transport. Alternative explanations, including reduced feed intake at higher doses or physiological impairment in stressed larvae, cannot be excluded.

A possible functional analogy is the vertebrate Na^+^/I^−^ symporter (NIS), the key membrane protein responsible for active iodide uptake in the thyroid and small intestine (Portulano et al. 2014). In the rat intestine, NIS-mediated transport is a high-affinity, saturable process with a reported Michaelis constant (K_m_) for iodide of approximately 13.4 µM (Nicola et al. 2009). This analogy is offered as a hypothesis for future testing rather than as an interpretation of the present data; no NIS orthologue has been identified in *T. molitor*. Furthermore, intestinal NIS expression in vertebrates is downregulated by high dietary iodide (Nicola et al. 2009). This offers 1 possible parallel to the observed decline in BAF, although the present data cannot distinguish between transporter downregulation and simple saturation.

An alternative mechanism for iodine uptake, peroxide-dependent diffusion, has been identified in sea urchin larvae (Miller and Heyland 2013). This process, which involves the enzymatic oxidation of iodide followed by diffusion, is characterized by a linear, non-saturable relationship between environmental iodine concentration and influx, which is not the pattern observed here. Should a carrier-mediated system be confirmed in insects, the parallel with the vertebrate NIS would raise the question of evolutionary convergence between protostomes and deuterostomes—but this remains speculative.

The steep increase in frass iodine is consistent with passive overflow following saturation of intestinal uptake, although the present data cannot distinguish this from actively regulated excretion. Once the transporters are fully occupied, any additional ingested iodine cannot be absorbed and is passively passed through the digestive tract. This route is consistent with studies in ruminants where feces are the main route for excreting excess dietary iodine from macroalgae (Lind et al. 2023).

### A Practical Threshold for Biofortification

From a practical standpoint, the saturation model defines a threshold rather than a window. Below approximately 112 mg I/kg supplementation, larvae concentrate iodine above dietary levels; above it, an increasing proportion of the fortificant is excreted rather than incorporated. Because half-maximal accumulation is reached at *K*_m_ = 86 mg/kg, most of the attainable enrichment is achieved within a relatively narrow, low-dose window.

For example, raising supplementation from 100 to 300 mg/kg—a 3-fold increase—raised larval iodine only from 102.6 to 147.3 mg/kg, a 1.4-fold increase. This demonstrates that the majority of the additional, expensive fortificant is lost through excretion. This provides a defined target range for feed formulators aiming to produce iodine-enriched insect meal while avoiding both under-fortification and inefficient over-fortification. A full cost analysis would require data on KI price, substrate cost, and processing yield, which were outside the scope of this study (Tariq et al. 2025).

### Iodine Toxicity and the Constraints of the Target Species

The present study indicates a threshold for adverse effects in *T. molitor*, with observational effects at a supplemented dose of 1,000 mg/kg and extensive mortality at 2,000 mg/kg or higher, suggesting a relatively high tolerance to iodine in the insect itself. Comparisons with vertebrate tolerance thresholds should be made with caution, as iodine metabolism in insects lacks the thyroidal component that governs iodine handling in vertebrates. This high tolerance, however, must be contextualized against the much lower tolerance levels reported for the target species in the animal feed industry. In aquaculture, a dietary iodine concentration of only 129 mg/kg dry weight was found to induce colloid goitre, a sign of iodine toxicity, in Atlantic cod larvae (Penglase et al. 2013). Similarly, in poultry, dietary concentrations exceeding 350 mg/kg have been shown to negatively impact egg production (Lewis 2004), with more recent studies indicating that even 8 mg/kg from an organic source can impair the performance and health of laying hens (Sarlak et al. 2020).

This comparison reveals a critical insight for applied biofortification: the practical upper limit for iodine enrichment is not dictated by the insect’s own physiological tolerance but by the much lower tolerance of the end consumer. While *T. molitor* larvae accumulated iodine to approximately 147 mg/kg at the highest supplementation level without observable adverse effects (300 mg I/kg), formulating a feed ingredient with such levels could contribute to iodine toxicity in sensitive target animals like fish larvae or laying hens, particularly when the iodine contribution from other dietary components is considered. In practice, mealworm meal constitutes only 1 component of compound feed, and the total dietary iodine from all ingredients must be evaluated to determine safe inclusion levels. Therefore, a “target-limited fortification ceiling” exists, where the safe and effective level of enrichment is defined by the requirements of the target animal, not the maximum capacity of the production organism.

### Broader Implications for Sustainable Feed Production and Nutrition

The results of this study have broader implications for sustainable agriculture and nutrition. Iodine-biofortified *T. molitor* larvae represent a novel functional feed ingredient that simultaneously delivers high-quality protein (Hong et al. 2020) and a controlled dose of an essential micronutrient. In contrast to the direct supplementation of inorganic iodine salts (e.g. KI or KIO_3_) into compound feeds, the biological incorporation of iodine into insect biomass may offer several important advantages, including enhanced iodine stability, reduced leaching losses, improved bioavailability, and safer nutrient delivery within a complex biological matrix (Cakmak et al. 2020). However, the extent to which iodine is organically incorporated within larval tissues remains to be elucidated, as chemical speciation ultimately determines its metabolic availability in the target animal. These properties are particularly relevant in aquaculture, where iodine added directly to feed pellets may partially dissolve into the surrounding water, leading to unpredictable dosing, environmental loading, and reduced feed efficiency. The incorporation of iodine into larval tissues may provide a natural microencapsulation effect, allowing a more controlled release during digestion (Gonzali et al. 2017).

Iodine is critical for the health, metamorphosis, and survival of fish larvae, and precise dosing is required to avoid toxicity (Lall and Kaushik 2021); the relevance of the present findings to aquaculture is therefore bounded by the tolerance of the target species. At the highest supplementation level at which larvae remained visibly unaffected (300 mg/kg), larval iodine reached 147 mg/kg—already above the 129 mg/kg dry weight reported to induce colloid goitre in Atlantic cod larvae (Penglase et al. 2013). Inclusion levels of iodine-enriched mealworm meal would therefore need to be set by the requirement of the receiving animal, not by the enrichment capacity of the insect.

The approach also aligns with the principles of a circular bioeconomy: rearing *T. molitor* on agricultural or food-industry by-products (Lienhard et al. 2023) and using the enriched larvae as a feed ingredient converts low-value side-streams into a higher-value product, while the resulting frass can be used as a biofertilizer (Verardi et al. 2025).

Finally, this work contributes to a food-system-based solution for ensuring human iodine adequacy. As public health initiatives advocating for reduced salt consumption potentially diminish the impact of universal salt iodization, a future “iodine gap” may emerge (Mikláš et al. 2021). Enriching animal products through biofortified feeds is a proven strategy to create alternative dietary iodine sources, as seen in milk and eggs (Sarlak et al. 2020). This study extends that principle 1 step further up the food chain, creating an enriched feed ingredient that can ultimately enhance the iodine content of farmed fish or poultry destined for human consumption.

### Limitations of the Present Study

Several methodological limitations must be considered when interpreting these results.

First, each dietary treatment was represented by a single rearing container. The 3 subsamples analyzed per treatment quantify within-container and analytical variability but do not constitute independent biological replicates; formal statistical comparison among treatments was therefore not possible, and the study is best regarded as a dose series rather than a replicated experiment.

Second, quantification was based on a single calibration series, and matrix-specific limits of detection and quantification and recovery were not established for larval and frass matrices. Because of the small sample masses required to remain within the calibration range, values for the control and lowest supplementation level lay close to the limit of quantification and should be regarded as indicative. Frass iodine at supplementation levels of 300 mg/kg and above could not be quantified at all, so the excretion pattern is characterized only over the lower part of the dose range.

Third, larvae from the 3 highest supplementation levels were collected dead and could not be depurated. Their iodine concentrations may include gut contents and adhering frass and were therefore excluded from the dose–response model, which is consequently valid only over the range 0 to 1,000 mg I/kg. The behavior of the system above this range cannot be characterized quantitatively from the present data.

Several further limitations arise from the scope of the study. First, the study quantified total iodine but did not determine its chemical form within the larval tissues. It remains unknown whether the iodine is stored as inorganic iodide or is organically incorporated, for example, into iodotyrosine residues. The chemical species of iodine can significantly influence its biological activity, bioavailability, and potential toxicity (Espino-Vázquez et al. 2022). From a biochemical standpoint, iodine within insect tissues could plausibly occur in multiple forms, including freely circulating inorganic iodide in the hemolymph, weakly bound iodide associated with extracellular proteins, or covalently incorporated iodinated amino acids generated through oxidative iodination reactions. In marine invertebrates and echinoderm larvae, iodide has been shown to undergo peroxidase-mediated incorporation into tyrosine residues, forming mono- and di-iodotyrosines (Moreda-Piñeiro et al. 2011, Espino-Vázquez et al. 2022), suggesting that enzymatic iodination pathways are not restricted to vertebrate thyroid tissue. It is therefore conceivable that a fraction of the accumulated iodine in *T. molitor* may be protein-bound rather than present solely as free iodide. Conversely, if a substantial proportion of the measured iodine represents unbound inorganic iodide retained within the hemolymph or gut lumen, its effective nutritional value and metabolic stability in target animals may differ from that of organically incorporated iodine. In vertebrates, the nutritional impact of iodine depends strongly on its chemical speciation, as organified iodine is metabolically integrated, whereas excess inorganic iodide is more rapidly excreted (Moreda-Piñeiro et al. 2011, Romarís-Hortas et al. 2012). Second, the high iodine concentration measured in the larvae does not guarantee high bioavailability to the animal that consumes them. The bioavailability of minerals from feed ingredients can be influenced by many factors, including chemical form and the presence of antinutritional factors such as chitin (Finke 2007, Schiavone et al. 2017). Third, this study was conducted using a single basal diet under controlled laboratory conditions. The kinetics of uptake and excretion might differ in larvae reared on different substrates, particularly those containing goitrogenic compounds (e.g. glucosinolates), which are known to interfere with iodine metabolism in vertebrates (Mikláš et al. 2021). Finally, while adverse effects were observed, their physiological basis was not investigated. The role of iodine and thyroid-like hormones in insect endocrinology is not well understood, but there is evidence that insects can concentrate iodine and that vertebrate thyroid hormones can elicit physiological effects (Davey 2000), leaving the precise mechanism of iodine toxicity in *T. molitor* as an open question.

### Directions for Future Research

Several directions arise from this study. First, iodine speciation should be addressed. Methods such as HPLC-ICP-MS, applied in selenium studies (Ferrari et al. 2022), could clarify whether *T. molitor* larvae act as carriers of inorganic iodide or convert it into organic, bioavailable forms. Given the analytical constraints encountered here, future work should employ ICP-MS, which offers a wider dynamic range and would allow frass iodine to be quantified across the full dose range. Second, in vivo trials are needed to test the bioavailability of iodine-fortified larvae in animals such as trout or broilers, assessing digestibility, thyroid function, growth, and tissue iodine content. Third, transcriptomic analyses of larvae under low vs. high iodine diets could identify candidate iodide transporter genes for functional characterization. Finally, studies should explore iodine toxicity mechanisms, focusing on effects on tyrosine metabolism, cuticle sclerotization (Gorman and Arakane 2010), and oxidative stress markers, to determine parallels with known mechanisms of iodine excess toxicity in vertebrates (Leung and Braverman 2014).

## References

[ieag098-B1] Akaike H. 1974. A new look at the statistical model identification. IEEE Trans. Automat. Contr. 19:716–723. 10.1109/TAC.1974.1100705

[ieag098-B2] Baker DH. 2004. Iodine toxicity and its amelioration. Exp. Biol. Med (Maywood) 229:473–478. 10.1177/153537020422900604 15169965

[ieag098-B3] Bednář J , Röhling S, Vohnout S. 1964. Contribution to determining protein iodine in the blood serum. Česk. Farm. 13:203–209. 14181336

[ieag098-B4] Behmer ST. 2009. Insect herbivore nutrient regulation. Annu. Rev. Entomol. 54:165–187. 10.1146/annurev.ento.54.110807.090537 18764740

[ieag098-B5] Bílek R , Bednár J, Zamrazil V. 2005. Spectrophotometric determination of urinary iodine by the Sandell–Kolthoff reaction subsequent to dry alkaline ashing. Results from the Czech Republic in the period 1994–2002. Clin. Chem. Lab. Med. 43:573–580. 10.1515/CCLM.2005.100 16006251

[ieag098-B6] Cakmak I , Marzorati M, Van Den Abbeele P, et al 2020. Fate and bioaccessibility of iodine in food prepared from agronomically biofortified wheat and rice and impact of cofertilization with zinc and selenium. J. Agric. Food Chem. 68:1525–1535. 10.1021/acs.jafc.9b05912 31942799

[ieag098-B7] Copelotti E , Fihurska L, Zanzot A, et al 2025. Calcium-enriched substrates to improve the nutritional composition of mealworm. Ital. J. Anim. Sci. 24:2703–2719. 10.1080/1828051X.2025.2592715

[ieag098-B8] Davey KG. 2000. Do thyroid hormones function in insects? Insect Biochem. Mol. Biol. 30:877–884. 10.1016/S0965-1748(00)00061-8 10876133

[ieag098-B9] EFSA Panel on Nutrition, Novel Foods and Food Allergens (NDA Panel); Turck D, Bohn T, Castenmiller J. 2023. Safety of UV‐treated powder of whole yellow mealworm (*Tenebrio molitor* larva) as a novel food pursuant to Regulation (EU) 2015/2283. EFSA. 21:1–32. 10.2903/j.efsa.2023.8009 PMC10233460 37274457

[ieag098-B10] Espino-Vázquez AN , Rojas-Castro FC, Fajardo-Yamamoto LM. 2022. Implications and practical applications of the chemical speciation of iodine in the biological context. Future Pharmacol. 2:377–414. 10.3390/futurepharmacol2040026

[ieag098-B11] Ferrari L , Sele V, Silva M, et al 2022. Biofortification of selenium in black soldier fly (*Hermetia illucens*) prepupae reared on seaweed or selenium enriched substrates. JIFF 8:887–899. 10.3920/JIFF2021.0153

[ieag098-B12] Finke MD. 2007. Estimate of chitin in raw whole insects. Zoo Biol. 26:105–115. 10.1002/zoo.20123 19360565

[ieag098-B13] Flachowsky G , Franke K, Meyer U, et al 2014. Influencing factors on iodine content of cow milk. Eur J Nutr 53:351–365. 10.1007/s00394-013-0597-4 24185833

[ieag098-B14] Franke K , Schöne F, Berk A, et al 2008. Influence of dietary iodine on the iodine content of pork and the distribution of the trace element in the body. Eur J Nutr 47:40–46. 10.1007/s00394-007-0694-3 18193376

[ieag098-B15] Gonzali S , Kiferle C, Perata P. 2017. Iodine biofortification of crops: agronomic biofortification, metabolic engineering and iodine bioavailability. Curr. Opin. Biotechnol. 44:16–26. 10.1016/j.copbio.2016.10.004 27835794

[ieag098-B16] Gorman MJ , Arakane Y. 2010. Tyrosine hydroxylase is required for cuticle sclerotization and pigmentation in *Tribolium castaneum*. Insect Biochem. Mol. Biol. 40:267–273. 10.1016/j.ibmb.2010.01.004 PMC2854195 20080183

[ieag098-B17] Herzig I , Trávníček J, Kursa V, et al 2007. Content of iodine in broiler meat. Acta Vet. Brno 76:137–141. 10.2754/avb200776010137

[ieag098-B18] Hong J , Han T, Kim YY. 2020. Mealworm (*Tenebrio molitor* larvae) as an alternative protein source for monogastric animal: a review. Animals 10:2068. 10.3390/ani10112068 PMC7695176 33171639

[ieag098-B19] Jankauskienė A , Aleknavičius D, Andrulevičiūtė V, et al 2024. Nutritional composition and safety parameters of mealworms (*Tenebrio molitor*) reared on substrates derived from by-products. Appl Sci 14:2744. 10.3390/app14072744

[ieag098-B20] Keil C , Maares M, Kröncke N, et al 2020. Dietary zinc enrichment reduces the cadmium burden of mealworm beetle (*Tenebrio molitor*) larvae. Sci Rep 10:20033. 10.1038/s41598-020-77079-x PMC7674442 33208833

[ieag098-B21] Kröncke N , Grebenteuch S, Keil C, et al 2019. Effect of different drying methods on nutrient quality of the yellow mealworm (*Tenebrio molitor* L.). Insects 10:84. 10.3390/insects10040084 PMC6523706 30934687

[ieag098-B22] Lall SP , Kaushik SJ. 2021. Nutrition and metabolism of minerals in fish. Animals 11:2711. 10.3390/ani11092711 PMC8466162 34573676

[ieag098-B23] Leung AM , Braverman LE. 2014. Consequences of excess iodine. Nat Rev Endocrinol 10:136–142. 10.1038/nrendo.2013.251 PMC3976240 24342882

[ieag098-B24] Lewis PD. 2004. Responses of domestic fowl to excess iodine: a review. Br J Nutr 91:29–39. 10.1079/BJN20031017 14748936

[ieag098-B25] Lienhard A , Rehorska R, Pöllinger-Zierler B, et al 2023. Future proteins: sustainable diets for *Tenebrio molitor* rearing composed of food by-products. Foods 12:4092. 10.3390/foods12224092 PMC10670904 38002150

[ieag098-B26] Lind V , Opheim M, Sandvik JT, et al 2023. Iodine intake and excretion from sheep supplemented with macroalgae (*Laminaria hyperborea*) by-product. Front. Anim. Sci. 4:1213890. 10.3389/fanim.2023.1213890

[ieag098-B27] Machado I , Priede AS, Rodríguez MC, et al 2024. Bioaccessibility of trace elements and Fe and Al endogenic nanoparticles in farmed insects: pursuing quality sustainable food. Food Chem. 458:140229. 10.1016/j.foodchem.2024.140229 38944920

[ieag098-B28] Makkar HPS , Tran G, Heuzé V, et al 2014. State-of-the-art on use of insects as animal feed. Anim. Feed Sci. Technol. 197:1–33. 10.1016/j.anifeedsci.2014.07.008

[ieag098-B29] Mikláš Š , Tančin V, Toman R, et al 2021. Iodine concentration in milk and human nutrition: a review. Czech J. Anim. Sci. 66:189–199. 10.17221/167/2020-CJAS

[ieag098-B30] Miller AEM , Heyland A. 2013. Iodine accumulation in sea urchin larvae is dependent on peroxide. J. Exp. Biol. 216:915–926. 10.1242/jeb.077958 23155081

[ieag098-B31] Moreda-Piñeiro A , Romarís-Hortas V, Bermejo-Barrera P. 2011. A review on iodine speciation for environmental, biological and nutrition fields. J. Anal. At. Spectrom. 26:2107. 10.1039/c0ja00272k

[ieag098-B32] Nicola JP , Basquin C, Portulano C, et al 2009. The Na^+^/I^−^ symporter mediates active iodide uptake in the intestine. Am. J. Physiol. Cell Physiol. 296:C654–C662. 10.1152/ajpcell.00509.2008 PMC2670652 19052257

[ieag098-B33] Nielsen TS , Engelsmann MN, Hansen SV, et al 2022. Bioavailability of different zinc sources in pigs 0–3 weeks post-weaning. Animals 12:2921. 10.3390/ani12212921 PMC9655132 36359046

[ieag098-B34] Peng D , Li Y-X, Dong L-X, et al 2024. Dietary iodine can effectively alleviate the adverse effects of fermented rapeseed meal on the growth, liver health, and antioxidant capacity of tilapia (GIFT, *Oreochromis niloticus*). Fishes 9:501. 10.3390/fishes9120501

[ieag098-B35] Penglase S , Harboe T, Sæle Ø, et al 2013. Iodine nutrition and toxicity in Atlantic cod (*Gadus morhua*) larvae. PeerJ. 1:e20. 10.7717/peerj.20 PMC3628846 23638355

[ieag098-B36] Plzák J , Svoboda M, Bednář V. 2009. Spectrophotometric determination of total iodine in environmental samples after alkaline mineralization. Chem. Pap. 2009:443–449.

[ieag098-B37] Portulano C , Paroder-Belenitsky M, Carrasco N. 2014. The Na+/I − symporter (NIS): mechanism and medical impact. Endocr. Rev. 35:106–149. 10.1210/er.2012-1036 PMC3895864 24311738

[ieag098-B38] Romarís-Hortas V , Bermejo-Barrera P, Moreda-Piñeiro J, et al 2012. Speciation of the bio-available iodine and bromine forms in edible seaweed by high performance liquid chromatography hyphenated with inductively coupled plasma-mass spectrometry. Anal. Chim. Acta 745:24–32. 10.1016/j.aca.2012.07.035 22938602

[ieag098-B39] Sarlak S , Tabeidian SA, Toghyani M, et al 2020. Supplementation of two sources and three levels of iodine in the diet of laying hens: effects on performance, egg quality, serum and egg yolk lipids, antioxidant status, and iodine accumulation in eggs. Ital. J. Anim. Sci. 19:974–988. 10.1080/1828051X.2020.1810142

[ieag098-B40] Schiavone A , De Marco M, Martínez S, et al 2017. Nutritional value of a partially defatted and a highly defatted black soldier fly larvae (*Hermetia illucens* L.) meal for broiler chickens: apparent nutrient digestibility, apparent metabolizable energy and apparent ileal amino acid digestibility. J Animal Sci. Biotechnol. 8:51. 10.1186/s40104-017-0181-5 PMC5465574 28603614

[ieag098-B41] Tariq MR , Liu S, Wang F, et al 2025. Black soldier fly: a keystone species for the future of sustainable waste management and nutritional resource development: a review. Insects 16:750. 10.3390/insects16080750 PMC12386371 40870551

[ieag098-B42] Van Der Fels-Klerx HJ , Camenzuli L, Van Der Lee MK, et al 2016. Uptake of cadmium, lead and arsenic by *Tenebrio molitor* and *Hermetia illucens* from contaminated substrates. PLoS One. 11:e0166186. 10.1371/journal.pone.0166186 PMC5112862 27846238

[ieag098-B43] Van Der Reijden OL , Zimmermann MB, Galetti V. 2017. Iodine in dairy milk: sources, concentrations and importance to human health. Best Pract. Res. Clin. Endocrinol. Metab. 31:385–395. 10.1016/j.beem.2017.10.004 29221567

[ieag098-B44] Verardi A , Sangiorgio P, Della Mura B, et al 2025. *Tenebrio molitor* frass: a cutting-edge biofertilizer for sustainable agriculture and advanced adsorbent precursor for environmental remediation. Agronomy 15:758. 10.3390/agronomy15030758

